# Isoform-specific roles of SIRT2 in regulating mitochondrial gene expression

**DOI:** 10.1093/geroni/igaf122.2545

**Published:** 2025-12-31

**Authors:** Xiaomin Zhang, Jyotsna Shrivastava, Gohar Azhar, Jeanne Wei

**Affiliations:** University of Arkansas for Medical Sciences, Little Rock, Arkansas, United States; University of Arkansas for Medical Sciences, Little Rock, Arkansas, United States; University of Arkansas for Medical Sciences, Little Rock, AR, LITTLE ROCK, Arkansas, United States; University of Arkansas for Medical Sciences, Little Rock, AR, LITTLE ROCK, Arkansas, United States

## Abstract

Sirtuin-2 (SIRT2), a member of the NAD+-dependent deacetylase family, is a key regulator of cellular processes such as metabolism, stress response and aging. We hypothesize that SIRT2 isoforms (v1, v2, v3) exert isoform-specific regulatory effects on mitochondrial-related genes, influencing mitochondrial dynamics, energy metabolism, and cellular homeostasis. To investigate this, SH-SY5Y cells, from a neuroblastoma cell line were transfected with empty vector (EV) or SIRT2 isoforms (v1, v2, v3), and the expression of mitochondrial genes, including Complex I subunits (NDUFAB1, NDUFV1, NDUFV2, NDUFS1), mitochondrial biogenesis and energy metabolism regulators (PGC-1α, PGC-1β), and mitochondrial dynamics and morphology markers (MFN-1, MFN-2, FiS1), were analyzed using quantitative RT-PCR (QuantStudio 3 Real-Time PCR System). Results revealed that SIRT2-v1 significantly upregulated mRNA levels of NDUFAB1 (p < 0.001), NDUFV1 (p < 0.01), and NDUFS1 (p < 0.05) compared to v2 and v3. SIRT2-v1 also increased PGC-1α expression (p < 0.05), numerically increased PGC-1β expression too and MFN-2 (p < 0.01), which are vital for mitochondrial biogenesis and mitochondrial fusion. These findings highlight the isoform-specific roles of SIRT-2 in modulating mitochondrial gene expression, with SIRT2-v1 predominantly influencing Complex I and PGC-1α. This study provides insights into the distinct regulatory effects of SIRT-2 v1 isoforms on mitochondrial function and metabolic pathways, underscoring their potential therapeutic relevance in mitochondrial-associated disorders. Additionally, the differential age-related increase in SIRT2 isoforms warrants further investigation.

